# Cardiac Failure and Cardiogenic Shock: Insights Into Pathophysiology, Classification, and Hemodynamic Assessment

**DOI:** 10.7759/cureus.72106

**Published:** 2024-10-22

**Authors:** Stavroula A Siopi, Polychronis Antonitsis, Georgios T Karapanagiotidis, Georgios Tagarakis, Christos Voucharas, Kyriakos Anastasiadis

**Affiliations:** 1 Cardiovascular Medicine, Aristotle University of Thessaloniki, Thessaloniki, GRC; 2 Cardiothoracic Surgery, Aristotle University of Thessaloniki, Thessaloniki, GRC

**Keywords:** acute heart failure, cardiogenic shock, hemodynamics, left heart failure, pressure-volume loop, right heart failure

## Abstract

Heart failure is defined as increased intracardiac pressures, either alone or combined with reduced cardiac output. Clinically, it is presented with signs and symptoms of congestion and compensated perfusion. Cardiogenic shock, on the other hand, is the spectrum of hemodynamic disturbances that lead to hypoperfusion or need for circulatory support, due to cardiac disease. Both entities affect millions of people worldwide, have a dismal prognosis, and constitute a severe socioeconomic burden. Heart failure can be the aftermath of ischemic heart disease, hypertension, arrhythmias, or cardiomyopathies. It undergoes multiple classifications, facilitating its investigation and treatment. The pathogenetic mechanisms differ in various types of heart failure, regarding the affected ventricles, the duration of symptoms, and their primary/secondary onset. These mechanisms reflect the complex interactions between cardiopulmonary, vascular, and hepatorenal systems. Acute deterioration of cardiac function can lead to cardiogenic shock. Myocardial infarction accounts for 81% of such cases. Healthy lifestyle and timely management of coronary artery disease are paramount, as they can prevent this life-threatening situation and reduce mortality and the economic burden for healthcare systems. Irrespective of the etiology, cardiogenic shock is interpreted using the pressure-volume loop. This can be modified for each ventricle, the underlying pathophysiology, and the time since symptoms' onset. It therefore provides valuable information about the native circulation and the expected alterations under mechanical or pharmacological support, facilitating the decision-making progress. In 2019, given the phenotypical heterogeneity of cardiogenic shock, the Society for Cardiovascular Angiography and Interventions introduced a classification system. According to this, patients are stratified in five stages proportionally to the severity of their condition. Aside from this classification, various biochemical, imaging, and hemodynamic monitoring indices are used to assess coagulation pathway and cardiac, hepatorenal, and pulmonary function, enabling the heart team to tailor therapy. Additionally, the prognostication progress is facilitated by scores, such as the Observatoire Regional Breton sur l'Infarctus (ORBI) score, the intra-aortic balloon pump (IABP) SHOCK-II score, and the CardShock score, indicating suitable escalation or de-escalation strategies. Despite the current progress, there are several areas of advancement regarding the role of vasoactive drugs in cardiogenic shock, revascularization options, mechanical ventilation patterns, hypothermia treatment, and mechanical circulatory support protocols.

## Introduction and background

Heart failure (HF) constitutes a clinical syndrome associated with symptoms (dyspnea/orthopnea, nocturnal polyuria, fatigue) and signs (edema, jugular vein distention, rales) of congestion and inadequate cardiac function [1-3]. On the other hand, the acute compromise of cardiac function, with clinical signs and symptoms of congestion and/or hypoperfusion, constitutes acute heart failure (AHF) [4]. AHF can be effectively treated with full recovery of cardiac function, lead to chronic heart failure (CHF), or deteriorate into cardiogenic shock (CS). CS is defined as the spectrum of hemodynamic disturbances in patients suffering from cardiovascular disease that lead to critical hypoperfusion or to the need for circulatory support to maintain hemodynamic stability [5].

From a pathophysiological perspective, HF is identified as the increase in intracardiac pressures, either alone or accompanied by low cardiac output (CO). It occurs at rest or during exercise and originates from structural or primary/secondary functional heart disease [3]. The most common cardiac causes of HF involve the myocardium (differentiation between systolic, diastolic, and combined HF) as well as the endocardium, pericardium, valvular mechanism, and conduction system [2,6]. Notwithstanding, the heart's ability to pump blood and the prognosis of HF are greatly affected by the arterial and venous vasculature. According to an extensive meta-analysis of 125 international studies from 1990 to 2020, the incidence of HF is estimated at 8.3% in adults over 65 years of age or lower among younger populations. The one-year mortality rate since the diagnostic confirmation is 33% regarding patients over 65 years, with minimal deviation among different age groups [7].

HF can be classified as right HF (RHF), left HF (LHF), or biventricular HF (pressure-volume overload of the right, left, or both ventricles, respectively). Based on the time of symptoms' onset, it is categorized as acute and chronic. Considering the ejection fraction (EF), it is classified as HF with preserved EF (HFpEF (EF>50%)), HF with mildly reduced EF (HFmrEF (40%<EF<49%)), and HF with reduced EF (HFrEF (EF<40%)). This classification applies to both acute and chronic HF [8-11].

Acetylcholine (ACH) most commonly is the result of an acute derangement of CHF. However, it may also arise from a de novo deterioration of the heart's pumping ability [12]. AHF can be further divided into the following: (i) hypertensive AHF, when systolic arterial pressure (SAP)>140 mmHg (the most common form), (ii) normotensive AHF, when 90 mmHg<SAP<140 mmHg, and (iii) hypotensive AHF, when SAP<90 mmHg [13]. The in-hospital mortality rate of AHF/CS remains high, ranging between 30% and 40% according to numerous reported studies [1,14].

Despite constant progress, the mean annual cost per HF patient remains remarkably high, namely, €4411 in Greece, €25532 in Germany, $10832 in the USA, $8089 in Japan, and $2128 in Nigeria. Worldwide, the mean annual cost of HF is approximately $108 billion [15-17], while according to a systematic review of 16 international studies, the estimated overall lifetime cost per patient is $127,000 [18]. These costs are linked with hospitalizations, rehabilitation, medication, and outpatient visits. They establish HF as an onerous economic burden and result in lower funding for the management of other ailments with high prevalence, such as coronary artery disease, renal failure, pulmonary disease, or cancer. Furthermore, these costs are expected to rise, given the demographic changes (increasing number of patients older than 65 years of age) and comorbidities. For these reasons, prudent use of resources, through the implementation of tailored and effective treatment strategies, is mandatory. 

This review article sheds light on HF and CS, diseases with dismal prognoses, that are integrated into everyday medical practice. It handles the classification, epidemiology, and pathophysiology of these entities, providing a broad spectrum of valuable knowledge. Moreover, in the CS section, a thorough analysis of hemodynamics, monitoring indices, staging, and prognostication scores, is presented, facilitating cardiologists and cardiac surgeons in the apprehension of complex principles. Lastly, the reader is informed about the current medical and mechanical treatment options in CS and future research perspectives into revascularization strategies in CS patients with multivessel coronary artery disease, the use of pharmacological agents (dopamine, norepinephrine, levosimendan, and serelaxin), mechanical ventilation, hypothermia therapy, and mechanical circulatory support (MCS) (type of device, time of initiation, and implementation protocols).

Methodology

We searched MEDLINE and Google Scholar databases for literature until April 2024, using the following keywords in different combinations: "acute heart failure", "biventricular heart failure", "cardiogenic shock", "cardiogenic shock guidelines", "cardiogenic shock monitoring", "cytokines in cardiogenic shock", "de novo heart failure", "economic burden of heart failure", "heart failure", "heart failure aetiology", "pathophysiology of cardiogenic shock", "pressure-volume loop", "prognostication scores in cardiogenic shock", "right heart failure after lvad", and "SCAI classification". The titles and abstracts of the results were then assessed, based on the eligibility criteria. The selected 123 articles were subjected to full-text analysis and included in this narrative review.

Eligibility Criteria

Articles pertaining to the pathophysiology, classification, epidemiology, and etiology of HF and CS, as well as to monitoring indices and prognostication scores in CS, were considered suitable for full-text analysis. Articles in languages other than English and articles referring to non-adult populations were excluded.

## Review

Cardiac failure

Pathophysiology of Primary LHF

Left ventricular (LV) dysfunction causes myocardial injury, as well as unfavorable vascular changes and neurohormonal activation. Firstly, the inability of the LV myocardium to contract effectively leads to decreased CO, end-organ hypoperfusion, and hypotension. Reactive tachycardia attempts to maintain normal stroke volume (SV). Simultaneously, it increases the oxygen demand of the already frail myocardium, worsening its perfusion and therefore its function. Secondly, the activation of the sympathetic plexus induces systemic vasoconstriction and increases the systemic blood pressure, in order to preserve coronary and systemic flow. Capacitance veins store one-fourth of the total blood volume, compensating for potential fluctuations in blood volume. In hypertensive AHF though, ventricular-vascular coupling fails, leading to increased pre- and afterload and causing further myocardial strain [19]. As a result, the rising pressure gradually reduces SV, leading to end-organ damage and ongoing cardiac ischemia. Lastly, these mechanisms trigger the release of inflammatory agents, such as reactive oxygen species, complement factors, tumor necrosis factor-alpha, and interleukins 1β, 6, 8, and 10. These mediators result in unfavorable vasodilation, while peroxynitrate and nitric oxide radicals additionally cause direct cardiac toxicity [5,6].

Right Cardiac Response to Primary LHF

Both ventricles normally produce equal SVs. The ineffective contraction of the LV increases the pressure load in pulmonary circulation and therefore the right ventricular (RV) afterload. Right cardiac chambers effectively counterbalance volume overload. However, unlike left chambers, they cannot adjust to pressure overload, due to their relatively thin myocardial walls, greater volume, and elasticity [20,21]. Consequently, RV function is impaired through various mechanisms. These include increased afterload secondary to pulmonary congestion (LV backward failure) and decreased preload, due to diminished CO (LV forward failure). Additionally, induced ischemia worsens the RV perfusion status (due to coronary hypoperfusion) and therefore the RV contractility. Primary LHF results in LV remodeling and shift of the ventricular septum rightwards. It therefore decreases systolic driving pressure in the RV, hampering its function. Finally, hypertrophy, dilatation, and restricted filling of the RV are caused by its compression into the nonelastic pericardium [22,23]. The ongoing RV dilatation displaces the ventricular septum towards the left, constraining the LV and worsening its already compromised function. These changes result in systemic and coronary hypoperfusion and further exacerbate this vicious cycle [24].

Right Cardiac Response to Left Ventricular Assist Device (LVAD)

In the case of LVAD implantation, the rightward displacement of the ventricular septum and the increase in RV preload can force uncoupling between the RV and the pulmonary artery (PA). Consequently, this leads to RV dilatation, tricuspid valve insufficiency, and a leftward shift of the septum, reducing RV SV and LV filling [25-27]. RHF occurs more frequently after the implantation of older, pulsatile LVADs. With the newer-generation, continuous-flow LVADs, the incidence is approximately 9-40% [28,29]. The main risk factors associated with RHF include the need for an intra-aortic balloon pump (IABP) or other temporary LVAD prior to permanent LVAD implantation, increased pulmonary resistance, and the use of LVAD as destination therapy [30]. Sildenafil, a phosphodiesterase-5 inhibitor, has been proposed as a prophylactic measure against secondary RHF during mechanical left cardiac support, with the hypothesis that it improves right heart function by reducing pulmonary resistance. However, the evidence supporting the success of this intervention remains weak [31].

Pathophysiology of Primary RHF

RHF is defined as reduced RV EF and compromised blood flow with either elevated or normal filling pressures at rest or during hyperdynamic circulation [32]. The most common cause of RHF is increased afterload, due to either left cardiac or pulmonary disease [23]. In such cases, the hypertrophy of the right cardiac chambers is followed by contraction abnormalities and an increase of chambers' volume. The ventricular septum is therefore shifted leftwards and entrains the tricuspid valve's tensor apparatus, leading to tricuspid valve insufficiency and secondary blood volume overload [33]. Other causes include conditions that disrupt the conduction system or interfere with RV contraction, such as right coronary artery stenosis or occlusion, arrhythmogenic RV cardiomyopathy, or cardiac injury, all of which reduce SV [34]. Lastly, systemic diseases or low left ventricular stroke volume (LVSV) can alter the RV preload, further impairing cardiac function [35]. The mortality rate of acute RHF, which is usually the aftermath of acute LHF or myocardial dysfunction (left or right), is estimated at 6-14% [36]. The manifestation of right heart dysfunction is a strong prognostic factor in CS [24].

Left Cardiac Response to Primary RHF

In primary RHF, the underlying cause involves structures from the lungs and backwards. LV preload is significantly decreased, resulting in reduced LVSV, SAP, and systemic and coronary blood flow. The primary cause is the prolongation of RV systole, leading to severely shortened RV diastole and reduced RV and LV filling rates [37]. The shortened RV ejection time and SV result in lower LV preload and CO [38]. Moreover, the hypertrophy or dilatation and remodeling of the RV shift the ventricular septum leftwards, further constricting the LV [39,40]. The previous mechanisms induce dyssynchrony in RV motion and RV-LV contraction, affecting RV wall stress, LV end-diastolic volume (EDV), and eventually SV [41,42]. It has been observed that in cases of acute ischemia, due to right coronary artery obstruction, RV dysfunction affects LV contractility. Furthermore, left cardiac dysfunction is being restored after RV reperfusion [43]. This phenomenon is the result of the distribution of common myocardial fibers in both ventricles, oriented from the epicardial to endocardial layers and spanning from the LV to the RV [24,44,45]. Disordered contractility of these common fibers, observed in right coronary obstruction, proposes another pathogenetic mechanism for LHF development secondary to primary RHF of ischemic origin.

Primary Biventricular AHF

In primary biventricular HF, the ability to eject blood is diminished in both ventricles simultaneously. This condition can be seen during cardiac tamponade, where fluid is accumulated between the nonelastic parietal and visceral pericardium. Consequently, filling pressures and sympathetic tone are increased, while diastolic filling of the atria and ventricles is impaired. These alterations decrease CO and eventually lead to the equalization of diastolic pressures across all cardiac chambers [46,47].

De Novo AHF vs. Acute Decompensation of CHF

De novo AHF is associated with higher in-hospital mortality (8.1%) compared to the acute decompensation of CHF (5.8%), as CHF provides adequate time so that the cardiopulmonary, vascular, and hepatorenal systems adjust to the new circumstances. Interestingly, the five-year mortality of acute-on-chronic HF surpasses that of de novo AHF (75.6% and 44.4%, respectively). Acute decompensation of CHF is more often related to underlying conditions (hypertension, coronary artery disease/unstable angina, diabetes mellitus, arrhythmias, stroke), while de novo AHF is associated with ST-segment elevation myocardial infarction (STEMI) and non-ST-segment elevation myocardial infarction (NSTEMI) [14,48-50]. Lastly, patients with acute decompensated CHF predominantly present with symptoms of congestion, whereas those with de novo AHF present with pulmonary edema and CS [51]. Understanding the pathophysiological interactions between LV and RV enables clinicians to timely identify compromised ventricular function that would otherwise be overlooked. They then can perform the appropriate adjustments in circulatory support and reduce mortality. A common scenario includes the development of RHF secondary to temporary LV mechanical support (usually with IABP or Impella). Symptoms of RHF could easily be neglected if not highly suspected, as they are rather vague and nonspecific. In this case, the deterioration of the patient's status could improperly be interpreted as the need for more robust LV support, thus worsening RHF and even leading to death.

CS

Terminology, Epidemiology, and Underlying Causes

Over the years, various studies have sought to define CS. The diagnosis of CS is based on the presence of hypotension, which is refractory to volume replacement, and evidence of hypoperfusion, such as altered mental status, cold extremities, and oliguria [52-57]. Secondary to that, hemodynamic indices such as low cardiac index (CI) and usually elevated pulmonary capillary wedge pressure (PCWP) can confirm the diagnosis. The SHOCK (1999) and IABP-SHOCK II (2012) trials are acknowledged as the cornerstones of CS studies (Table 1).

**Table 1:** Clinical characteristics of CS according to major published studies References: [53,54,56,58,59] The clinical criteria proposed by these major studies allow clinicians to immediately identify patients suffering from or at increased risk for CS. They can therefore timely initiate circulatory support and then proceed with the secondary laboratory and hemodynamic assessment. CI: cardiac index; CS: cardiogenic shock; LV: left ventricular; PCI: percutaneous coronary intervention; PCWP: pulmonary capillary wedge pressure; SAP: systemic arterial pressure; LVEF: left ventricular ejection fraction

Study (year), [reference]	Definition of CS
SHOCK (1999), [53]	SAP<90 mmHg for >30 min or inotropes to maintain SAP>90 mmHg, end-organ hypoperfusion (urine output<30 mL/h or cold extremities), hemodynamic assessment: CI<2.2 L/min/m^2^ and PCWP>15 mmHg
IABP-SHOCK II (2012), [54]	SAP<90 mmHg for >30 min or catecholamines to maintain SAP>90 mmHg, clinical indications of pulmonary congestion, end-organ hypoperfusion (disorientation, livedo reticularis, urine output<30 mL/h, serum lactates>2 mmol/L, cold extremities)
ESC-HF Guidelines (2016), [56]	SAP<90 mmHg after adequate fluid administration, clinical (cold extremities, oliguria, disorientation, pulsus mollis) and laboratory (metabolic acidosis, high serum lactates, high serum creatinine) indications of end-organ damage
TRIUMPH (2007), [58]	Patency of infarct-related artery spontaneously or after PCI, refractory CS>1 h after PCI with SAP<100 mmHg despite vasopressors (dopamine≥7 μg/kg/min or epinephrine≥0.15 μg/kg/min), clinical or hemodynamic criteria for elevated LV filling pressure, end-organ hypoperfusion, LVEF<40%
CULPRIT-SHOCK (2017), [59]	Planned early revascularization by PCI, multivessel coronary artery disease defined as >70% stenosis in at least two major vessels (≥2 mm diameter) with identifiable culprit lesion, pulmonary congestion, SAP<90 mmHg for >30 min or inotropes to maintain SAP>90 mmHg, indications of end-organ damage (altered mental status, cold/clammy skin and extremities, urine output<30 mL/h, serum lactates>2 mmol/L)

The SHOCK registry studied the effect of emergency revascularization on 30-day and six-month mortality rates in acute myocardial infarction-related cardiogenic shock (AMICS) patients. It proved significant mortality reduction in six months and established the emergency revascularization as the foundation of AMICS management [53]. The IABP-SHOCK II trial studied the impact of IABP as a circulatory support method in patients with AMICS and proved no benefit from its use. Both studies have profoundly changed the guidelines and recommendations, regarding CS treatment options.

Patients suffering from CS, AHF, or CHF are classified into four groups according to their volume load (euvolemic, hypervolemic) and the CO (sufficient, insufficient). A simpler phenotypical approach is to differentiate patients according to their volume status (dry or wet) and perfusion status (warm or cool) [57,60]. In this classification, "warm and wet" patients have the best prognosis while "cold and dry" the worst [61,62]. Euvolemic patients are more likely to suffer from chronic kidney disease or have experienced a myocardial infarction (MI) [63], whereas the "wet and warm" phenotype is more frequently linked to systemic inflammatory response syndrome (SIRS) and MI [52,64]. The "cold and wet" type is the most frequent subtype in AMICS, accounting for approximately 64% of cases. These patients typically present with low CI and elevated systemic vascular resistance and PCWP. The second most common phenotype is "cold and dry" (28%), followed by "wet and warm" (6%) and "wet and dry" (3%) [6,63]. Regarding therapeutic targets, the "warm and dry" patients can benefit from the optimum management of the underlying cardiovascular disease. The "warm and wet" phenotype requires vasodilation in case of fluid redistribution and fluid reduction through diuretics or renal replacement therapy. The "cold and dry" patients need adequate fluid administration and inotropic agents. Finally, treatment of the "cold and wet" patient includes inotropes, vasodilators, and diuretics in case of normal systolic blood pressure (SBP) or vasopressors in case of refractory hypotension and hypoperfusion [6]. A CI>2.2 L/min/m^2^ does not exclude CS, as in various cases a preserved CI is accompanied by end-organ hypoperfusion, due to microvascular dysfunction [65,66]. Moreover, CS can result from acute RV dysfunction, LV dysfunction, or biventricular dysfunction, each associated with different characteristics and treatment strategies (Table 2).

**Table 2:** Basic hemodynamic characteristics of CS, originating from RV dysfunction, biventricular dysfunction, and LV dysfunction References: [67,68] PAPi is calculated as the ratio of pulmonary artery pulse pressure (pulmonary artery systolic pressure-pulmonary artery diastolic pressure) to CVP and serves as an indirect indicator of RV function. CS: cardiogenic shock; CVP: central venous pressure; LV: left ventricular; PAPi: pulmonary artery pulsatility index; PCWP: pulmonary capillary wedge pressure; RV: right ventricular

Variables	RV dominant CS	Biventricular CS	LV dominant CS
CVP (mmHg)	>14	>14	<14
PCWP (mmHg)	<18	Varying	>18
CVP/PCWP	>0.86	>0.86	<0.86
PAPi	<1.5	<1.5	>1.5

The 12-month mortality of CS is nearly 50% [52,53,69,70]. The most common cause is acute MI (81%), with STEMI leading to CS twice as often as NSTEMI [52], followed by decompensated HF, post-cardiotomy shock, ventricular outflow tract obstruction, post-cardiorespiratory arrest shock, stunned myocardium after septic shock or post-SIRS, myocardial contusion, valvular heart disease, conduction disturbances, and, finally, non-cardiac systemic and pharmacologic causes of obstruction (Table 3) [5,71]. 

**Table 3:** Etiology of CS References: [5,71] CS: cardiogenic shock; CHF: chronic heart failure; SIRS: systemic inflammatory response syndrome

Affected cardiac structure in CS	CS underlying disease
Myocardium	Acute myocardial infarction, right ventricular infarction, acute decompensation of CHF, cardiomyopathies/pregnancy-related cardiac disorders, toxins/endocrinological and metabolic disturbances, post-cardiotomy shock, myocardial depression due to SIRS, myocardial contusion/free wall rupture/papillary muscle rupture/ventricular septal rupture, hypothermia
Valvular	Native valve stenosis/insufficiency, obstruction, prosthetic valve-related mechanical failure
Conduction system	Supraventricular tachycardia, ventricular tachycardia, bradycardia, advanced atrioventricular blocks
Vascular/extra-cardiac structures	Pulmonary embolism, tamponade, tension pneumothorax, acute aortic syndrome

The Hemodynamics of CS

**Figure 1:**
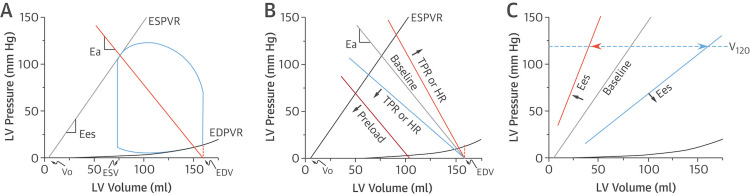
Pressure-volume loops and relations in normal cardiac function Reference: [73]; reprinted with permission Ea: arterial elastance; EDPVR: end-diastolic pressure-volume relationship; EDV: end-diastolic volume; Ees: end-systolic elastance; ESPVR: end-systolic pressure-volume relationship; ESV: end-systolic volume; HR: heart rate; LV: left ventricular; TPR: total peripheral resistance

**Figure 2:**
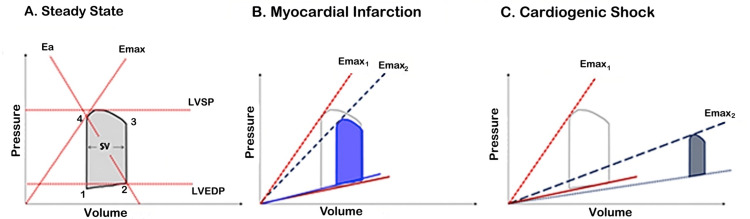
LV pressure-volume loop in normal cardiac function (A) in comparison to the acute phase of myocardial infarction (B) and to CS (C) Reference: [74]; reprinted with permission LV: left ventricular; Ea: arterial elastance; Emax: maximal elastance; LVEDP: left ventricular end-diastolic pressure; LVSP: left ventricular systolic pressure; SV: stroke volume; CS: cardiogenic shock

Irrespective of the underlying etiology, the pathophysiology and hemodynamic assessment of CS are interpreted using the pressure-volume loop (PV loop). The shape of a PV loop under normal conditions is depicted in Figure 1A and Figure 2A and is dependent on both preload and afterload [72-74].

Each side of the loop represents one phase of the cardiac cycle as follows: (1) isovolumic contraction, (2) ejection, (3) isovolumic relaxation, and (4) ventricular filling. The width of the loop represents SV (EDV-end-systolic volume (ESV)=SV), while the height represents end-systolic pressure (ESP). The loop conforms to the end-systolic pressure-volume relationship (ESPVR) and the end-diastolic pressure-volume relationship (EDPVR) axes. The ESPVR represents ventricular relaxation when all the actin-myosin bonds are uncoupled. It increases in conditions such as hypertrophic or constrictive cardiomyopathy and infiltrating diseases and decreases in dilated cardiomyopathy and ventricular remodeling.

The EDPVR defines the passive diastolic properties or stiffness of the ventricle, representing the interaction between pressure and volume load (dP/dV), and increases simultaneously with end-diastolic pressure (EDP). ESPVR intersects the PV loop at a certain point. The slope of the line extending from this point to the EDV corresponds to arterial elastance (Ea). It is proportional to total peripheral resistance (TPR) and heart rate (HR) and equal to the ratio of ESP and SV. Ea represents the "Windkessel effect" of vascular walls, which is the conversion of mechanical energy produced during the systole into kinetic energy, eventually transforming pulsatile SV into continuous blood flow. The ESPVR's slope is represented by the end-systolic elastance (Ees) value (maximal elastance (Emax) in Figure 2), which is equal to the ratio of ESP to ESV and indicates ventricular contractility. When Ees is increased, systolic pressure values correspond to lower filling volumes (anticlockwise shift), while when Ees is decreased, the opposite is observed (clockwise shift). The Vo volume is the maximum ventricular unstressed volume, while V120 is the volume that correlates with an LV pressure value of 120 mmHg. These findings suggest that elastance changes have almost no impact on Vo and vice versa, Ees is independent of volume and ventricular contractility factor (Figure 1C). The intersection of ESPVR and Ea represents the equilibrium between pressures, flows, contractility, and HR with SV, a phenomenon known as ventricular-vascular coupling. This can also be defined as the ratio of Ea to Ees (Figure 1B) [75,76].

The area defined by the blue loop represents the stroke work (SW), which is defined as the product of mean arterial pressure (MAP) and SV. Cardiac power output (CPO) represents cardiac performance and serves as an important prognostic factor in CS, indicating the product of SW that the heart can generate externally [72]. Optimal SW generation is achieved when Ea/Emax=1. Myocardial oxygen consumption (MVO_2_) per stroke represents the necessary amount of oxygen to support the baseline metabolism, the transcellular transport of calcium ions, and the actin-myosin coupling and uncoupling. In other words, it represents the amount of oxygen needed for the actual product of SW. Changes in HR interfere with the MVO_2_ per minute, but not with the MVO_2_ per stroke. MVO_2_ per stroke is proportional to the area bounded by the ESPVR, EDPVR, and systolic fragment of the PV loop and is equal to the summation of SW and potential energy (PE). Furthermore, PE is defined by the ESPVR, EDVR, and diastolic fragment of the loop and represents the myofilaments' residual energy at the end of the contraction that was not deployed to produce work (Figure 1a) [77].

In the case of acute MI, the disorder accounting for most CS cases, the contractility of the affected ventricle is significantly decreased (lower Ees=Emax). This results in a proportional decrease of the slope of ESPVR, pressure, SV, and CO with a slight increase in EDP and PCWP (Figure 2B). Initially, baroreceptors in the great vessels detect the decrease of MAP and activate the efferent autonomic cardiac and vascular neural fibers. Consequently, catecholamines are being released, increasing HR, contractility, and TPR, as well as elevating central and pulmonary venous pressure. Moreover, they increase the effective circulating blood volume, by shifting blood from high-capacitance reservoirs in the splanchnic vasculature to low-capacitance central vessels. These neurohormonal adaptations lead to a further downward and rightward shift of the PV loop, towards higher EDPs and EDVs. Simultaneously, many inflammatory mediators are also being produced. Cytokines such as interleukins 1β, 6, 7, 8, and 10 and tumor necrosis factor-alpha reduce TPR, counteracting the previously presented adaptation mechanisms, while interleukin 1β is additionally involved in cardiac fibrosis. Peroxynitrate and nitric oxide radicals cause unfavorable vasodilation and direct cardiac toxicity, through the inhibition of myocardial contractility, suppression of mitochondrial respiration, disturbance of glucose metabolism, and reduced catecholamine responsiveness [78]. Moreover, C5a recruits neutrophils, which infiltrate the myocardium and produce cardiotoxic proteolytic enzymes. Ischemia leads to endothelial cell injury and cellular calcium overload, which generates reactive oxygen species and cell necrosis [5,6,79]. Overall in AMICS, ESPVR is closer to the volume axis, the PV loop is narrower, shorter, and transported rightwards, and EDPVR is slightly elevated (Figure 2C). Contractility, SV, and MAP values are diminished, while TPR varies from decreased to elevated [72,80,81].

**Figure 3:**
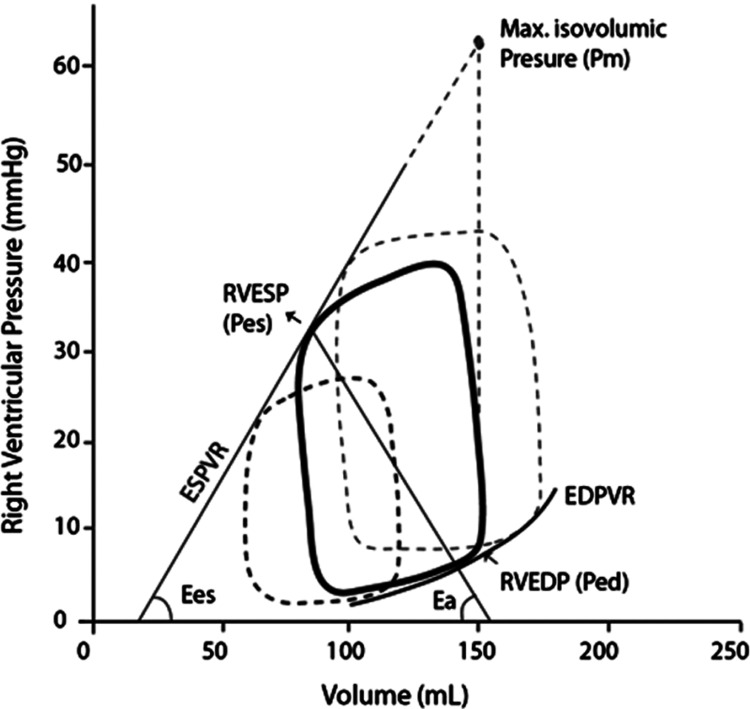
Pressure-volume loop of the RV in normal cardiac function Reference: [82]; reprinted with permission Ea: arterial elastance; EDPVR: end-diastolic pressure-volume relationship; Ees: end-systolic elastance; ESPVR: end-systolic pressure-volume relationship; RV: right ventricular; RVEDP: right ventricular end-diastolic pressure; RVESP: right ventricular end-systolic pressure

The RV PV loop has a narrower Ees (estimated to be one-fifth to one-seventh of the LV value) and decreased systolic pressures (Figure 3). EDP and EDV are equal in both ventricles, due to their approximately equal sizes. Furthermore, in the RV loop, the isovolumetric fragments are not clearly defined, unlike in LV, as diastolic pulmonary pressure is decreased and almost equal to the EDP of RV. Finally, the RV PV loop has a bell-like shape in normal cardiac function, while after MI it resembles the LV loop, being shifted rightwards and becoming narrower and shorter [24,72]. Fundamental for the right cardiac function is the coupling of the RV, which represents energy transfer and depends on Ees and Ea, with an Ees/Ea ratio of approximately 2 indicating optimal right cardiac function. In cases of thickened RV myocardium during remodeling, contractility-to-load coupling remains unaffected. However, in maladapted remodeling, when dilatation of RV occurs, coupling fails and RHF evolves [82,83].

Compromised RV function can be the aftermath of a disorder initially affecting right cardiac chambers, leading to primary RHF, or it can result from LHF or LVAD implantation, known as secondary RHF. The pathophysiology of these two entities is being discussed in sections "Pathophysiology of Primary RHF", "Right Cardiac Response to Primary LHF", and "Right Cardiac Response to Left Ventricular Assist Device (LVAD)", respectively.

CS Staging

The currently used classification of CS was proposed in 2019 by the Society for Cardiovascular Angiography and Interventions (SCAI). It was developed in order to create clusters of patients exhibiting similar phenotypes, considering the heterogeneity of the disease. It therefore simplifies the assessment of each group and standardizes the treatment strategies for these patients. SCAI CS classification can be broadly applied in emergency departments, catheterization laboratories, or intensive care units and includes the following five stages (Table 4).

**Table 4:** SCAI CS classification References: [74,84] BD: base deficit; BNP: B-type natriuretic peptide; CI: cardiac index; CVP: central venous pressure; GFR: glomerular filtration rate; HF: heart failure; HR: heart rate; JVP: jugular venous pressure; MAP: mean arterial pressure; MI: myocardial infarction; PA: pulmonary artery; PCWP: pulmonary capillary wedge pressure; SCAI: Society for Cardiovascular Angiography and Interventions; SAP: systolic arterial pressure; CS: cardiogenic shock

Stage	Presentation	Signs and symptoms	Biochemical indices	Hemodynamics
A (at risk)	No symptoms, history of MI or HF	Normal peripheral pulse, JVP, alertness, pulmonary auscultation	Normal serum lactates, renal function, and remaining blood test values	SAP>100 mmHg or baseline, CI>2.5 L/min/m^2^, CVP≤10 mmHg, PCWP≤15 mmHg, PA saturation≥65%
B (beginning)	Hemodynamic instability without hypoperfusion	Elevated JVP, normal peripheral pulse and alertness, fine crackles	Normal serum lactates, elevated BNP, first-degree renal decompensation	SAP<90 mmHg, MAP<60 mmHg or fall>30 mmHg from baseline, HR>100 bpm, normal remaining parameters
C (classic)	Hypotension, fatigue	Altered mental status, cold and clammy skin, coarse crackles, >2s capillary refill time, urine output<30 mL/h	Serum creatinine>1.5× baseline or >0.3 mg/dL or GFR<50%, lactates>2 mmol/L, elevated BNP	CΙ<2.2 L/min/m^2^, PCWP>15 mmHg
D (decline)	Declining, failed treatment	As in stage C but declining	As in stage C but declining	Dose increase or vasopressor addition or mechanical support
E (extremis)	Collapse/ongoing cardiopulmonary resuscitation	Loss of consciousness, cardiac collapse, multiple defibrillations	Lactates≥8 mmol/L, pH<7.2, BD>10 mEq/L	Persistent hypotension despite maximum support, vasopressors bolus intravenously

Stage A/at risk for CS: These patients are stable and do not meet the criteria for pre-shock (stage B) or shock stages (stages C-E), but have a diagnosis of acute MI, AHF, or acute decompensation of CHF.

Stage B/beginning of CS: These patients maintain normal perfusion (CI>2.2 L/min/m^2^, normal mentation, serum lactates, serum creatinine, and hourly urine output) with evidence of hemodynamic instability, such as hypotension (MAP<60 mmHg or fall>30 mmHg from baseline) or reactive tachycardia (HR>100 bpm). They require volume substitution or redistribution.

Stage C/classic CS: Patients in stage C present with hypoperfusion (CI<2.2 L/min/m^2^, elevated lactates and/or serum creatinine, and/or decreased hourly urine output), but successfully respond to initial pharmacologic or mechanical support, with potential minor modifications.

Stage D/deterioration of patient's status: There is an unsuccessful response to initial treatment or a need for an intensive approach. They require one or more additional vasoactive agents or MCS, to maintain hemodynamic stability.

Stage E/extremis: The patient is unconscious and in cardiac collapse/arrest. Refractory shock is present, despite high doses of vasoactive agents and the implementation of MCS.

Hypoperfusion is described as the inadequate end-organ blood supply. It is manifested as a decrease in glomerular filtration rate and hourly urine output, an increase in serum creatinine and lactates, and a deterioration in alertness. Hypotension is the decreased systolic and/or MAP (SAP<90 mmHg and/or MAP<60 mmHg) and constitutes a very common hemodynamic index. Systemic arterial blood pressure depends on CO and systemic vascular resistance, while perfusion is dependent on arterial blood pressure and regional vascular resistance. Therefore, hypotension has organ-specific effects on oxygenation and can be isolated or combined with hypoperfusion [85]. Patients in stage B preserve normal perfusion status but are hemodynamically unstable (pre-shock stage). In stage C, hypoperfusion is the main characteristic, which may or may not be accompanied by hypotension. The patients who present with hypoperfusion, even with normotension, face a higher mortality risk. It is worth mentioning that there have been objections regarding the criteria for stages B and C, as some suggest that patients in stage B should exhibit hypoperfusion with normal or low SAP, while those in stage C should have hypoperfusion, hypotension, and successful response to initial treatment [86].

The need for significant circulatory support with potential low-impact changes classifies one patient into stage C. If hemodynamic stability cannot be achieved with initial measures, the patient is classified into stage D. Finally, if stabilizing a patient in CS is futile and hypoperfusion persists, they are classified into stage E. The need for higher doses of vasopressors results in advancement within the SCAI staging system. A patient who achieves hemodynamic stability with the implementation of two types of vasopressors in low doses is considered a stage C patient, while a patient requiring a single agent at high doses is classified into stage D. In more complex cases, the combination of the number of supportive methods and the dose of the medical agents should be considered during the staging process [87].

Assessment of circulatory sufficiency using serum lactates should be performed cautiously, as lactate levels may not always correlate with hemodynamic status in various situations (e.g., CHF, mesenteric ischemia, compartment syndrome, or end-organ hypoperfusion with normal serum lactates). In such cases, additional parameters, such as EF and CI, should be evaluated. Generally, serum lactates>2 mmol/L classify the patient into stage C or higher. Regardless of the given stage, cardiac arrest constitutes an independent prognostic factor and should be considered only when patients are unable to respond to verbal commands or have a Glasgow Coma Scale score of <9 after resuscitation and not in brief episodes followed by complete neurological recovery [88].

The use of SCAI staging has limitations. It is recommended as only one component of assessment and should always be used in conjunction with the underlying cause of CS (especially in ischemic vs. non-ischemic CS) and the type of decompensation (de novo AHF or decompensation of CHF). Potential pitfalls in the use of the SCAI staging system can be avoided, with rigorous monitoring, timely invasive hemodynamic assessment, and constant implementation of prognostication scores indicated for each SCAI stage. Lastly, age and comorbidities significantly impact prognosis and treatment selection strategies. Nonetheless, they are not considered independent factors [89].

Monitoring

The diagnosis of CS is achieved through the application of the SCAI staging system. Several additional serum biomarkers are proposed for the assessment of CS. High-sensitivity troponin typically follows a rise-and-fall pattern due to acute ischemic injury but may maintain lower values in cases of delayed patient arrival, in the presence of hibernating or stunned myocardium. The typical rise-and/or-fall pattern is absent in the case of CS of non-ischemic etiology, where troponin is elevated, reflecting myocardial strain or damage, caused by disorders other than acute coronary syndromes (ACS) [90]. B-type natriuretic peptide (BNP) levels are elevated and associated with increased mortality in AMICS. It is of note though that their value should always be interpreted considering the underlying disease, as they can be increased in disorders such as sepsis-related AHF or RHF, without constituting a CS assessment index. Elevated levels are also observed for high-sensitivity C-reactive protein, soluble tumor necrosis factor receptor-1, endothelin-1, and procollagen III N-terminal peptide [91]. Blood oxygenation and respiratory function are assessed through respiratory rate, pulse oximetry, and serum lactates, which are typically elevated [92]. Moreover, mixed venous oxygen saturation (SvO_2_) represents the end result of oxygen consumption and delivery, through the measurement of oxygen saturation of blood sample, obtained from the superior vena cava, inferior vena cava, and coronary sinus. Normal SvO_2_ values range between 65% and 70%, with higher ones being found in sepsis, hypothermia, or hyperoxia and lower ones in anemia, hypoxia, hyperthermia, or CS. The fluctuations of these values, along with arterial blood gas analysis, provide a comprehensive monitoring of the tissue oxygenation [5]. SAP and central venous pressure should also be continuously monitored, and their assessment should rely not only on single measurements but also on the overall trends.

Hypoperfusion due to pump failure can potentially lead to acute kidney injury and vice versa, renal failure could indicate end-organ hypoperfusion. Therefore, the renal function should be evaluated through serum creatinine and hourly urine output, simple indices widely used in everyday clinical practice. Notably, newly introduced markers cystatin C, neutrophil gelatinase-associated lipocalin, and kidney injury molecule-1 have not been proven superior to serum creatinine [93].

Liver function is assessed using aspartate aminotransferase, alanine aminotransferase, lactate dehydrogenase, serum bilirubin, and prothrombin time. In order to differentiate liver congestion due to the acute decompensation of right CHF, from CS-related liver dysfunction, the ratio of alanine aminotransferase to lactate dehydrogenase can be used, with values <1.5 indicating hepatic injury as a result of CS [5].

The coagulation pathway can be disturbed in various ways, in the setting of CS. The most common ones are the use of oral anticoagulant or/and antiplatelet therapy, as well as the administration of unfractionated heparin, potentially inducing thrombocytopenia (heparin-induced thrombocytopenia syndrome). Decreased levels of vitamin K in case of hepatic dysfunction lead to lower serum levels of factors II, VII, IX, and X. Lastly, when MCS is implemented, the external coagulation pathway is activated, due to blood contact with the elements within the circuits. Coagulation is evaluated using prothrombin time-international normalized ratio, activated partial thromboplastin time, thrombin time, activated coagulation time, anti-Xa levels, and, when necessary, thromboelastography [5].

In addition to biomarkers, chest X-rays can provide insight into cardiac size, aortic wall status, pulmonary parenchymal status, and correct positioning of the endotracheal tube, catheters, cannulae, and pacemaker leads. In cases of suspected aortic disease or pulmonary embolism, further examination with computed tomography scanning is strongly advised. A 12-lead electrocardiogram (ECG) provides information regarding ischemia, pulmonary embolism, conduction abnormalities, myocarditis, and electrolyte disturbances or intoxication. The status of the central nervous system is assessed using the Glasgow Coma Scale and optic nerve sheath diameter measurement [5]. Notably, these means should not be used as diagnostic tools for CS, as the diagnosis is based on the presence of hypoperfusion or hemodynamic stability that requires circulatory support. They can either be used as prognostication tools, when integrated into CS scoring systems, or provide information regarding various organ functions and the origin of CS (e.g., enlarged cardiac silhouette in chest X-rays could imply cardiomegaly, found in cardiac tamponade, and ST-segment elevation in ECG indicates that the origin of CS is acute MI).

Cardiac assessment is completed using transthoracic and transesophageal echocardiography [5]. It is noteworthy that the suitability of the LVEF as a representative indicator of cardiac function and a prognostic factor has been questioned, as the mortality rate reported in prospective cohort studies was similar among patients classified in different grades of HF based on LVEF [94]. It is currently accepted that LVEF does not provide insights into the underlying cause. Additionally, it does not accurately reflect ventricular contractility, as in many cases such as in diabetes mellitus, diabetic patients proposed an increased risk of death or development of cardiac failure, despite their allegedly preserved EF [95,96]. Moreover, LVEF is influenced by preload, afterload, and various cardiovascular comorbidities [96-98]. A different echocardiographic indicator, global LV longitudinal strain, is proven superior to LVEF in predicting major adverse cardiac events. It is considered more reliable for cardiac assessment in HFpEF or ACS, due to its increased sensitivity for wall strain and ischemia [99-104].

Prognostication Scores

Many clinical and laboratory parameters have been introduced to facilitate the assessment of patients suffering from CS and applied treatments [72,105-115]. Patients are divided into two groups: the pre-shock group (stages A-B) and the classic-shock group (stages C-E) (Table 5).

**Table 5:** Prognostication scores from major CS trials References: [105-107,110-115] ACC-NCDR: American College of Cardiology-National Cardiovascular Data Registry; ACS: acute coronary syndrome; ALKK: Arbeitsgemeinschaft leitende kardiologische Krankenhausärzte; CABG: coronary artery bypass grafting; CS: cardiogenic shock; FMC: first medical contact; HR: heart rate; LVEF: left ventricular ejection fraction; ORBI: Observatoire Regional Breton sur l'Infarctus; PCI: percutaneous coronary intervention; SAP: systolic arterial pressure; SCAI: Society for Cardiovascular Angiography and Intervention; STEMI: ST-segment elevation myocardial infarction; TIA: transient ischemic attack; TIMI: thrombolysis in myocardial infarction

Trial (year), [reference]	Parameters	Participants	SCAI stage
Obling et al. (2018), [110]	Age, stroke, symptom onset to intervention, anterior STEMI, HR/SAP ratio, comatose status after cardiopulmonary resuscitation	2247 STEMI patients treated by primary PCI	A-B
ORBI (2018), [107]	Age>70 years (2 points), previous stroke/TIA (2 points), cardiac arrest upon admission (3 points), anterior STEMI (1 point), FMC-to-PCI delay>90 min (2 points), Killip class (2-6 points), combination of SAP<125 mmHg and pulse pressure<45 mmHg (4 points), glycemia>10 mmol/L (3 points), culprit lesion in the left main artery (5 points), post-PCI TIMI-flow<3 (5 points)	6838 patients without CS upon admission and treated by primary PCI	A-B
ALKK (2004), [111]	Left main artery disease, TIMI-flow<3 post-PCI, advanced age, three-vessel disease, long time intervals between symptom onset and PCI	1333 patients from the ALKK registry	C-E
Sutton et al. (2005), [112]	Previous myocardial infarction, age>70 years, failed reperfusion	113 patients	C-E
ACC-NCDR (2005), [113]	Age, female gender, serum creatinine>2 mg/dL, total occlusion of the left anterior descending artery, no stent used, no glycoprotein IIb/IIIa inhibitor used	483 patients from the ACC-NCDR	C-E
TRIUMPH (2009), [114]	SAP, creatinine clearance, number of vasopressors used, norepinephrine dose used	396 patients from the TRIUMPH trial	C-E
SHOCK (2010), [115]	Clinical score: age, anoxic brain damage, end-organ hypoperfusion, shock upon admission, prior CABG, non-inferior infarction, serum creatinine≥1.9 mg/dL, SAP hemodynamic plus clinical score: stroke work, LVEF<28%, age, anoxic brain damage, end-organ hypoperfusion	1217 patients from the SHOCK trial and registry	C-E
CARD-SHOCK (2014), [103]	Prior CABG, ACS etiology, confusion, previous infarction, serum lactates, LVEF, age, SAP	219 patients from a multicenter European registry	C-E
IABP-SHOCK II (2017), [106]	Age>73 years (1 point), previous stroke (2 points), blood glucose>191 mg/dL or 10.6 mmol/L (1 point), serum creatinine>1.5 mg/dL or 132.6 μmol/L(1 point), serum lactates>5 mmol/L (2 points), TIMI-flow<3 post-PCI (2 points)	600 patients from the IABP-SHOCK II study	C-E

Regarding the pre-shock group, the Observatoire Regional Breton sur l'Infarctus (ORBI) score is the best validated tool for predicting the development of classic CS after primary percutaneous coronary intervention (PCI). It derives from the ORBI trial, which studied the incidence and impact of high-degree atrioventricular block in patients with STEMI. The trial showed that patients with high-degree atrioventricular block had higher mortality rates. Based on 11 variables collected in the catheterization laboratory, the score stratifies patients into four groups (low risk 0-7 points, low to intermediate risk 8-10 points, intermediate to high risk 11-12 points, and high risk >13 points) and indicates the ones that are at risk for CS and would benefit from pharmacological or MCS [107]. 

For patients with evident CS (stages C-E), the most widely acknowledged score is the IABP-SHOCK II score, deriving from the IABP-SHOCK II trial. The score calculates 30-day mortality by assessing clinical, laboratory, and catheterization parameters and classifies patients into low (0-2 points), intermediate (3-4 points), and high (5-9 points) risk, associated with 23.8%, 49.2%, and 76.6% mortality rates, respectively [106,108,109]. This score presents considerable strengths. Firstly, the number of AMICS patients being studied in the IABP-SHOCK II trial was the largest among contemporary randomized clinical trials. Secondly, the variables used are simple and thus can be easily calculated in clinical practice. Thirdly, the variables are routinely obtained during cardiac catheterization and do not require further elaborated assessment. Lastly, it is validated not only in the IABP-SHOCK II trial but also in external CS cohorts. This score is similar among all CS patients, regardless of the potential use or type of MCS. It can therefore guide circulatory support, by determining 30-day mortality risk and indicating which patients would potentially benefit from escalation of therapy [116].

Finally, the CardShock study was designed to create a CS prognostication score, which calculates short-term mortality, regardless of CS etiology. The CardShock score estimates 12-day mortality, based on seven parameters, and classifies patients into low (0-3 points), intermediate (4-6 points), and high (7-9 points) risk categories, with 8.7%, 36%, and 77% mortality, respectively [72,105]. It is of note that the thresholds in risk classification and the examined parameters differ among the scoring systems (as shown in Table 5). The groups that emerge though reflect mortality rates, regardless of the prognostication score being used.

Scoring systems used for general intensive care unit patients, such as the Acute Physiology and Chronic Health Evaluation (APACHE) II/III/IV [117,118], Simplified Acute Physiology Score (SAPS) 2/3 [119], and Sequential Organ Failure Assessment (SOFA) [120] scores, can also be applied, with APACHE II and III and SAPS 2 scores being superior in predicting mortality [121].

In a CS cohort from the University Heart and Vascular Center Hamburg, the IABP-SHOCK II score was compared to the CardShock score. C-indices were similar among the two scoring systems, for patients suffering from CS. This result was also validated by the Mayo Clinic external validation cohort. In the latter, the APACHE III score showed the highest C-index among prognostication scores used in general intensive care units. Lastly, the general prognostication scores proposed lower C-indices, compared to scores designed specifically for CS assessment [122].

Therapeutic Strategies

The management of CS is multidimensional. Firstly, it evolves general supportive measures (arterial oxygen saturation>92%, normal arterial acid-base balance, serum lactates, electrolytes, and glucose, as well as rigorous monitoring of coagulation pathway and hepatic, renal, and thyroidal function). Secondly, emphasis should be placed on addressing the primary disorder. This includes primary PCI in most ACS cases, coronary artery bypass grafting, or fibrinolysis for the rest of ACS patients, percutaneous or surgical valve replacement/repair for severe valvopathies, or ablation treatments for arrhythmias. Thirdly, circulatory support with the help of pharmacological agents (vasoactive medications such as norepinephrine, dopamine, or vasopressin) and/or MCS (IABP, Impella, TandemHeart, extracorporeal membrane oxygenation (ECMO), or their combinations) should be initiated [109].

Future perspectives

A common issue that remains to be addressed is the revascularization strategies implemented in AMICS patients, presenting with multivessel coronary artery disease, as studies have shown benefit from the revascularization of both culprit and non-critical, non-culprit lesions. Another critical topic in CS management that requires further research is the use of optimal vasoactive medication. Though dopamine is a widely used agent, it is associated with an increased risk of arrhythmias, when compared to other inotropes/vasopressors. The Sepsis Occurrence in Acutely Ill Patients (SOAP) II trial compared the use of dopamine vs. norepinephrine in CS patients and proved that even though mortality rates were similar for both agents, dopamine was related to the aforementioned adverse events. On the other hand, newer medications, such as levosimendan and serelaxin, a vasodilator hormone targeting relaxin-2, studied in the Relaxin in Acute Heart Failure (RELAX-AHF)-II trial and showing no benefit of its use in CS management, need extensive assessment. Furthermore, the effects of mechanical ventilation on cardiac function in CS remain nebulous. Despite current knowledge regarding the cardiopulmonary interaction, the ideal ventilation mode (positive end-expiratory pressure level) is yet to be determined. A promising strategy for the management of CS is induced hypothermia. This intervention is based on the hypothesis that decreased core temperatures (32-34°) reduce oxidative stress and inflammation, thus protecting the myocardium. Nevertheless, the clinical impact of hypothermia in CS should be examined more thoroughly and established as a therapeutic tool in more CS centers [5,123]. Novel kidney indices, though non-superior to serum creatinine, should be better examined as potential prognostic factors for renal assessment, in the setting of CS, diabetes mellitus, or renal disease alone [93]. The role of nitrates and their cardiac implications, as well as optimal renal replacement therapies in the management of AHF, need to be clarified more precisely [56]. Finally, despite the plethora of information regarding MCS in CS, a broadly accepted protocol defining the type of initial mechanical support, optimal escalation and de-escalation strategies, and concomitant medication has not yet been established [5].

## Conclusions

AHF and CS are related to considerably high mortality rates that approximate 50%, due to hemodynamic deterioration, multiorgan dysfunction, or SIRS. The latter leads to unfavorable vasodilation, volume distribution abnormalities, and the release of cardiotoxic mediators, such as reactive oxygen species, peroxynitrate, and nitric oxide radicals. Early identification of cardiac decompensation is pivotal, as it enables the cardiac team to timely initiate circulatory or cardiac support and address the underlying disorder. Of great importance is the rigorous monitoring of vital functions and supporting parameters, to perform any required adjustments in mechanical and pharmacological therapy. The related prognostication scores facilitate decision-making progress, indicating escalation or de-escalation strategies, bridging towards transplantation or implantation of permanent ventricular assist devices. To satisfy these requirements, high expertise and collaboration between cardiologists, cardiac surgeons, intensivists, and perfusionists are necessary, and it is therefore recommended that patients suffering from AHF or CS are transported to a tertiary HF center as soon as hemodynamic stability is achieved or transportation is considered safe.

Ongoing advancements expand the medical armamentarium and substantially upgrade the currently available therapies. Despite the progress being made, the need for greater improvement in this demanding field persists. Future research should focus on the underlying pathogenetic pathways, prognostic indices, optimization of pharmacological and mechanical therapy, and systemic response to the applied methods. Healthcare providers will therefore be able to act promptly and with greater precision, thus reducing mortality and improving quality of life.
